# Diversity bias in colorectal surgery: a global perspective

**DOI:** 10.1007/s13304-022-01355-w

**Published:** 2022-09-09

**Authors:** Franco Marinello, Christina A. Fleming, Gabriela Möeslein, Jim Khan, Eloy Espín-Basany, Gianluca Pellino, Diversity Bias Study Collaborators, Yongbo An, Vittoria Bellato, Miguel Cunha, Nagendra N. Dudi-Venkata, Cristián Gallardo, Zoe Garoufalia, Gloria Zaffaroni, Nuha Yassin, Richard R. W. Brady, Peter Christensen

**Affiliations:** 1 Colorectal Surgery, Vall d’Hebron University Hospital, Universitat Autònoma de Barcelona UAB, Barcelona, Spain; 2 Department of Colorectal Surgery, CHU Bordeaux, Bordeaux, France; 3 Royal College of Surgeons in Ireland, Dublin, Ireland; 4 Center for Hereditary Tumors, Ev. Krankenhaus BETHESDA, University of Düsseldorf, Duisburg, Germany; 5 Colorectal Department, Portsmouth Hospitals University NHS Trust, Portsmouth, UK; 6 School of Health and Social Care, University of Portsmouth, Portsmouth, UK; 7 Department of Advanced Medical and Surgical Sciences, Università degli Studi della Campania “Luigi Vanvitelli”, Naples, Italy

**Keywords:** Diversity, Gender, Race, LGBTQ +, Colorectal surgery

## Abstract

There is a specific lack of data on equity and injustices among colorectal surgeons regarding diversity. This study aimed to explore colorectal surgeon’s lived experience of diversity bias with a specific focus on gender, sexual orientation or gender identity and race or religion. A bespoke questionnaire was designed and disseminated to colorectal surgeons and trainees through specialty association mailing lists and social media channels. Quantitative and qualitative data points were analysed. 306 colorectal surgeons responded globally. 58.8% (*n* = 180) identified as male and 40.5% (*n* = 124) as female. 19% were residents/registrars. 39.2% stated that they had personally experienced or witnessed gender inequality in their current workplace, 4.9% because of sexual orientation, and 7.5% due to their race or religion. Sexist jokes, pregnancy-related comments, homophobic comments, liberal use of offensive terms and disparaging comments and stereotypical jokes were commonly experienced. 44.4% (*n* = 135) did not believe their institution of employer guaranteed an environment of respect for diversity and only 20% were aware of society guidelines on equality and diversity. Diversity bias is prevalent in colorectal surgery. It is necessary to work towards real equality and inclusivity and embrace diversity, both to promote equity among colleagues and provide better surgical care to patients.

**Supplementary Information:**

The online version contains supplementary material available at 10.1007/s13304-022-01355-w.

## Introduction

In current practice, the medical and surgical community has become far more cognisant of the importance of diversity and the importance of promoting equality, diversity and inclusivity (EDI) in work practice. This has been a result of considerable reports of inequality, prejudice and discrimination that has been prevalent in the workforce. The experience of diversity bias is common in surgery with discrimination broadly reported against women, the “lesbian, gay, bisexual, transgender, and questioning” (LGBTQ +) community and discrimination based on race and religion as frequently described reasons [1]. While great progress has been made in reducing the prevalence of overt or explicit bias, the experience of unconscious or implicit bias is still prevalent [2]. Unconscious bias is commonly experienced as micro-aggressions. Micro-aggressions may be described as subtle snubs, ‘slights’ and insults directed towards an individual’s identity based on a stereotype or historic bias about the social or cultural group that they identify with [3].

As a traditionally male dominated profession, feminisation of the surgical workforce is increasing. However, women can experience a high rate of gender-based discrimination in medical school, residency and practice [4, 5]. Moreover, the synthesised literature of the lived experience of women in surgery suggests that gender bias both discourages female medical students from pursuing a career in surgery and contributes significantly to higher attrition [6]. There is also little data on diversity bias experienced by colleagues who identify as LGBTQ + , or those who belong to a minority ethnic race or religion. A recent report, showed these surgeons had endured one or more type of experience short of harassment, bullying or abuse in their workplace [7].

To date there is a lack of reported data on the experience of diversity bias specifically among colorectal surgeons. The aim of this work was to explore the colorectal surgeon’s (residents/registrars and attendings/consultants) lived experience of diversity bias with a specific focus on gender, race or religion and sexual orientation or gender identity.

## Materials and methods

### Questionnaire creation and validation

The aim of this questionnaire was to explore bias in colorectal surgery relating to three specific factors i.e., gender, sexual orientation, or gender identity (LGBTQ +), and race or religion. A semi-structured online questionnaire was used to collect data from the study respondents with the principle of snowball sampling method (non-probabilistic sampling) via “Google Form”, an online self-administered data collection and hosting tool (‘Google LLC’ Mountain View, CA, USA).

For developing the online questionnaire, two patterns were selected including: single answer questions through multiple choice options, and open questions through short answers. No sample size calculation was performed since this was a cross-sectional study. The questionnaire contained three sections. Section one included demographic characteristics of the respondents (age, gender, and country of professional practice and stage). Section two explored potential gender related inequalities experienced or witnessed at the respondent’s workplace. Section three explored potential bias related to the respondents or co-workers’ sexual orientation. Section four investigated possible prejudices related to race or religion of the respondents. For these sections, if the answer was positive, they were asked to further describe specific situations or experiences through multiple response options and/or through a brief free-text explanation. Section five investigated participant awareness of working place or scientific association policies or recommendations relating to diversity bias. Following initial design, the questionnaire was distributed and piloted among the steering group to ensure content and face validity. The feedback received from this was used to further refine the question items both in terms of content and wording to minimise ambiguity and improve question neutrality. The questionnaire is detailed in Appendix A. The design of the survey and the reporting of the results adhered to the CHERRIES Guidelines [8].

### Population identification and dissemination

Colorectal surgeons (consultants/attendings) and colorectal surgeons in training (resident/trainee/fellow) worldwide were invited to participate in this study. The questionnaire was opened for a six-week study period: July 1st to August 15th 2021. The questionnaire was disseminated through mailing lists of several European surgical scientific societies including: the Spanish Association of Coloproctology (AECP), the Italian Association of Colorectal Surgery (SICCR), the European Society of Coloproctology (ESCP), and the Association of Surgeons of Great Britain and Ireland (ASGBI). The questionnaire was further disseminated through social media platforms including Twitter and WhatsApp groups using the following link: “https://forms.gle/D85erYLQo9HRbHMDA “. The responses were anonymised and confidential in line with Google’s privacy policy (https://policies.google.com/privacy?hl=it, accessed on 2 January 2021). Participants were required to provide informed consent by clicking a specific checkbox at the end of the questionnaire, after being informed that all data would be used only for research purposes and that name and institution of practice could not be identified. Participation in the questionnaire was voluntary and no incentive was offered. Formal review board approval was not required due to the nature of the study.

### Analysis

Data collected and gathered in the google forms was downloaded in Microsoft excel sheet (Microsoft, Richmond, Virginia) for analysis. Quantitative variables were reported using frequency (percentage) whereas mean [standard deviation (SD)] was used to report quantitative variables. No further statistical analysis was performed given the sample characteristics. The qualitative comments were summarized for inductive thematic analysis. A list of areas for action was developed, based on the results of the survey.

## Results

### General characteristics

**Fig. 1:**
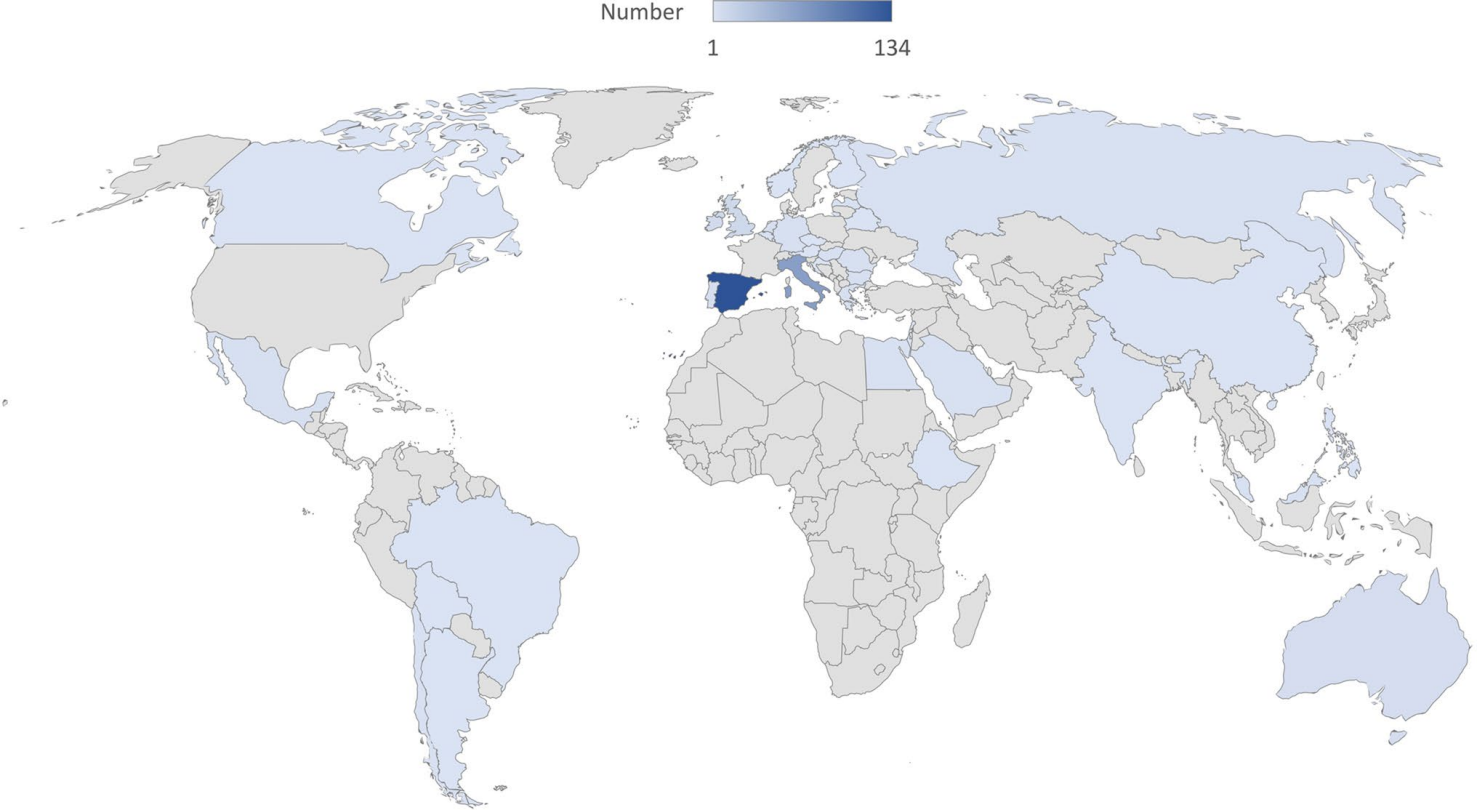
Heatmap of countries represented by respondents

A total of 306 participants responded to the questionnaire during the study period from 37 different countries. Countries that are represented are summarised in Fig. 1. This included 58.8% (*n* = 180) who identified as male and 40.5% (*n* = 124) who identified as female. The majority of responses were from consultant surgeons (*n* = 248–81%), and the rest from residents/trainees. The most common age group responding was the 36–45 years group (*n* = 111–36.3%). A complete summary of demographic characteristics and primary country of practice for all respondents are summarised in Table 1.

**Table 1:** Demographic information of participants

Total no. participants	% (*n*)
Gender	
Male	58.8 (180)
Female	40.5 (124)
Not specified	0.7 (2)
Age categories (years)	
25–35	25.5 (78)
36–45	36.3 (111)
46–55	26.5 (81)
56–65	9.5 (29)
> 65	2.2 (7)
Career stage	
Resident	19 (58)
Consultant	81 (248)
Primary Country of Practice	
Spain	43.8 (134)
Italy	20.9(64)
United Kingdom	3.3 (10)
Ireland	2.3 (7)
Australia	1.6 (5)
Other European Union Countries	10.1 (31)
Other Non-European Union Countries	8.2 (25)
Not specified	9.8 (30)
Specialty Association Memberships	
ACPGBI	11.4 (35)
AECP	35 (107)
ASGBI	5.2 (16)
ESCP	57.2 (175)
SICCR	18.6 (57)
Other National Colorectal or General Surgery society	32 (98)
No Scientific Society related to Coloproctology	7.2 (22)

### Gender-related bias

**Fig. 2:**
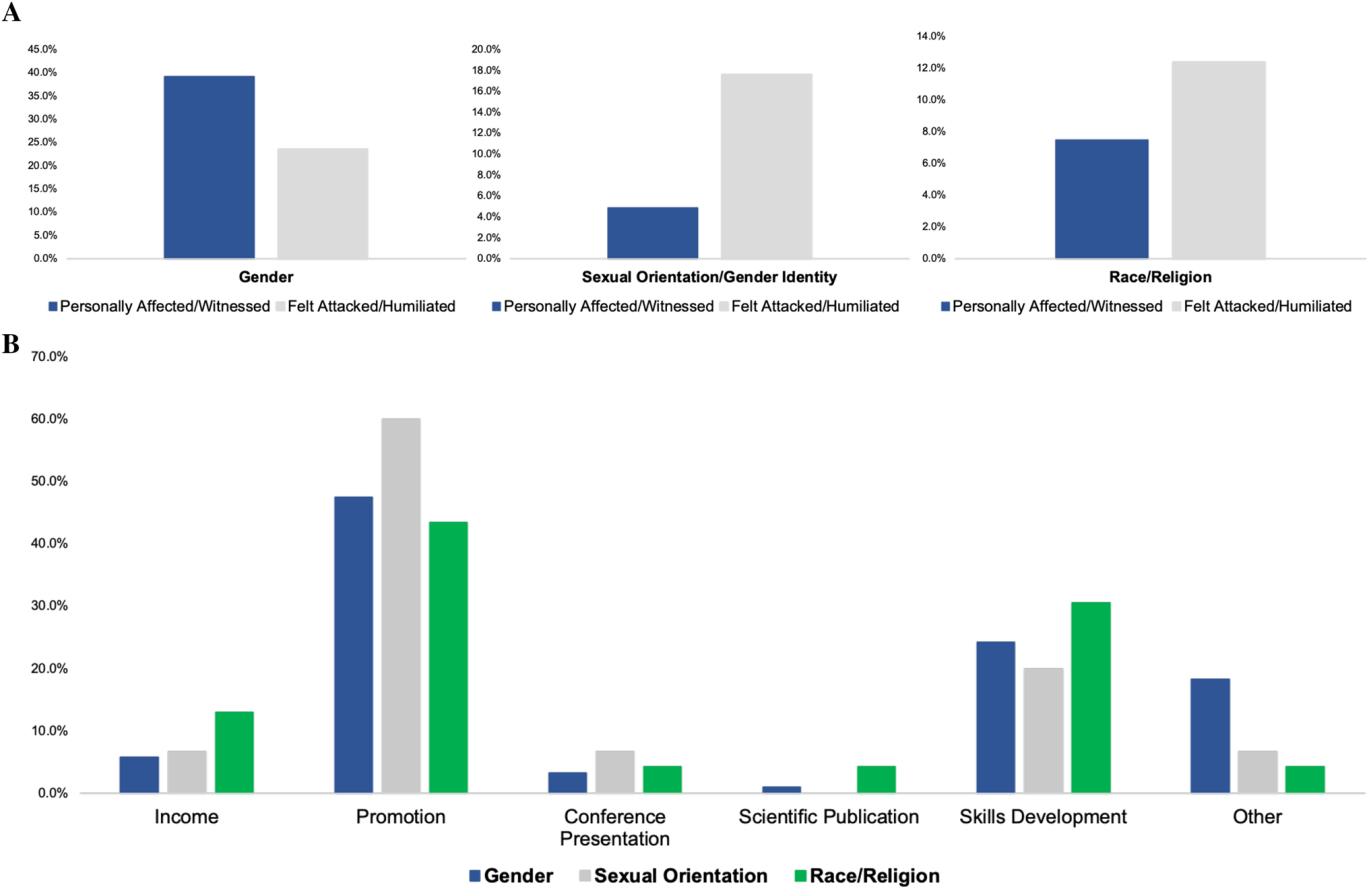
Summary of experience of diversity bias in current colorectal surgery practice (**A**) and professional areas that have been impacted by bias (**B**)

One-hundred and twenty respondents (39.2%) stated that they had personally experienced or witnessed first-hand situations of gender inequality in their current workplace (Table 2). The main categories are summarised in Table 3. Of them, the main inequalities were described as: not achieving a promotion or appropriate professional advancement (47.5%), lack of opportunity to develop a particular surgical technique (24.2%), and impact on income due to gender differences (18.3%). Some respondents also described that they felt they were not invited to present at conferences or attend congresses due to their gender (3.3%) (Fig. 2). Furthermore, 72 respondents (23.5%) reported that they had suffered attacks, humiliating comments, or a negative work environment due to their gender. The majority of these experiences were described as sexist jokes, or pregnancy-related comments (Table 3).

**Table 2:** Personal experiences of diversity bias in current practice and personal impact of diversity bias as reported by participants

Personal reported experiences of diversity bias in current practice		
	Overall group	
	Yes % (*n*)	No % (*n*)
Gender		
Personally affected/witness situations of gender inequality	39.2 (120)	60.8 (186)
Felt attacked/subjected to humiliating comments	23.5 (72)	76.5 (234)
Sexual orientation/gender identity		
Personally affected due to your sexual orientation	4.9 (15)	95.1 (291)
Felt attacked/subjected to humiliating comments	17.6 (54)	82.4 (252)
Race/religion		
Personally affected due to your race/religion	7.5 (23)	92.5 (283)
Felt attacked/subjected to humiliating comments	12.4 (38)	87.6 (268)

Personal reported impact of diversity bias			
% (*n* =)	Gender bias (*n* = 120)	Sexual orientation/gender identity (*n* = 15)	Race/religion (*n* = 23)
My income has been affected	5.8 (7)	6.7 (1)	13 (3)
Impact on promotion or professional advancement	47.5 (57)	60 (9)	43.5 (10)
Not invited to present/attend at congresses	3.3 (4)	6.7 (1)	4.3 (1)
Publishing in scientific journals	0.9 (1)	–	4.3 (1)
Development of surgical technique	24.2 (29)	20 (3)	30.6 (7)
Other	18.3 (22)	6.7 (1)	4.3 (1)

**Table 3:** Qualitative thematic summary of experience of harassment and experience of humiliating comments

	Qualitative themes (from free text responses)
Gender bias	General sexist behaviour and comments Inappropriate sexual comments “risk of pregnancy”…”rota gaps” Menstrual references to explain behaviour “this procedure needs a strong man” “the girl”, “good girl”, “not bad for a girl” “too….” – “ambitious, blond, emotional, difficult”
Sexual orientation/gender identity	Homophobic comments Use of derogatory terms Insinuation of HIV + status “not a real man”, “not manly enough”
Race/religion	General racist comments Racist “jokes” “dirty”, “poor”, “all the same” Anti-Islamic/terrorist characterisations Expected to perform lower resulting in less operative opportunity

### Sexuality-related bias

Only 15 respondents (4.9%) stated that they had been personally affected by their sexual orientation at their workplace (Table 2). The main experience categories described were not achieving a promotion or advancing professionally when expected (52.6%), lack of opportunity to develop a particular surgical technique (21.1%) and direct impact on income (15.8%) (Fig. 2). A total of 54 surgeons (17.6%) indicated that they experienced or witnessed attacks, humiliating comments, or a negative work environment because of their or other colleagues’ sexual orientation, mainly due to homophobic comments and jokes, or the liberal use of offensive terms (Table 3).

### Race and religion-related bias

Twenty-three (7.5%) responding colorectal surgeons stated that they had been personally affected by their race or religion in their workplace (Table 2). Of these experiences, the main categories again were not achieving a promotion or advancing professionally (38.5%), not achieving the opportunity to develop a particular type of surgical technique (26.9%), and impact on income (11.5%) (Fig. 2). Also, 38 respondents (12.4%) described that they experienced or witnessed attacks, humiliating comments, or a negative work environment because of their race or religion, especially due to disparaging comments or stereotypical jokes (Table 3).

### Institutions, specialty associations and call for change

**Fig. 3:**
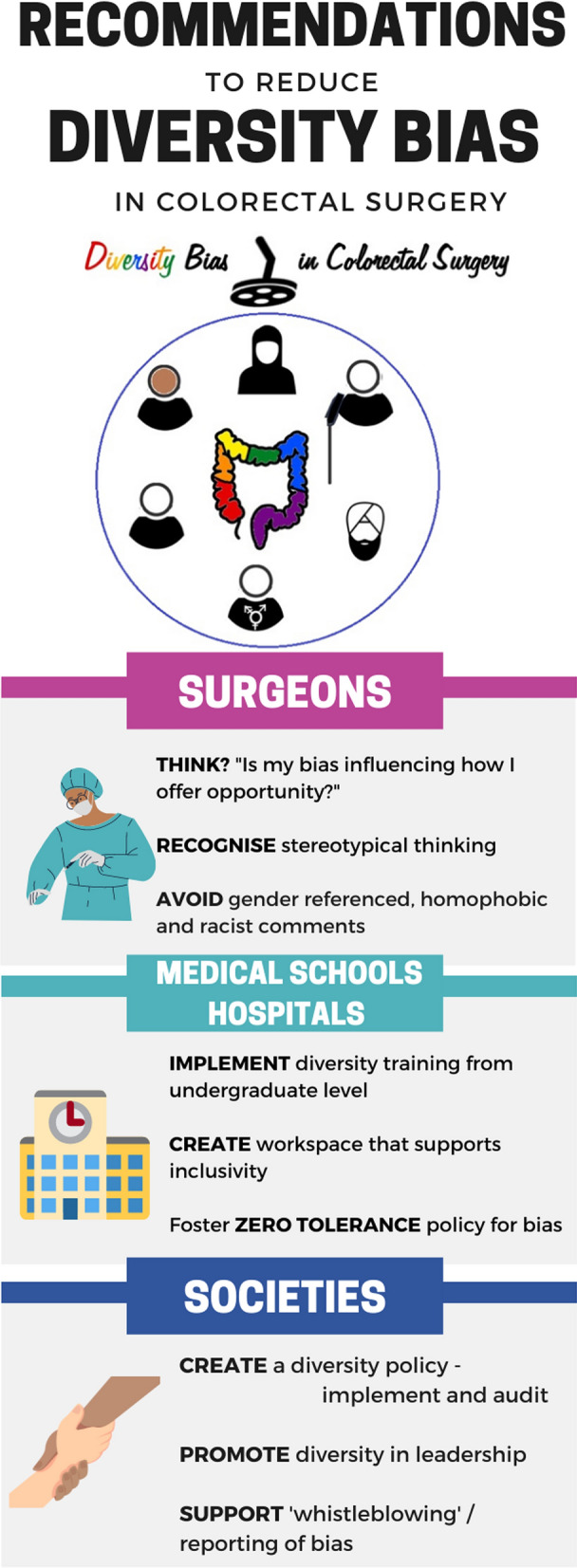
Infographic recommendations for surgeon, institution and specialty society level change based on results to overcome diversity bias in colorectal surgery

Almost half of the respondents (*n* = 135–44.4%) thought their work institution or employer did not guarantee respect in gender equality, sexual orientation, or race diversity. Furthermore, the majority of them (*n* = 252–82.9%) indicated their work institution or employer did not develop programs regarding respect in these topics. Only 20% of the colorectal surgeons who are affiliated with Scientific Societies, stated that the scientific association they hold membership with had a program regarding respect in gender equality, sexual orientation, or race diversity. In Appendix B, the authors outline a summary of all free-text comments describing the experience of diversity bias from respondents. From this data the experience of bias described in each situation could have been improved by surgeons in 88.6% (*n* = 70) of cases, medical schools, hospitals or institutions in 46.8% (*n* = 37) and speciality societies in 35.4% (*n* = 28). Based on the findings of this study, the authors recommend specific action plans to reduce diversity bias in colorectal surgery (Fig. 3).

## Discussion

This study identified that diversity bias is experienced in global colorectal surgery practice. Almost half of respondents had experienced or witnessed gender bias in their current workplace with, 4.9% experiencing bias due to sexual orientation or gender identity and 7.5% due to race or religion. The study further identified how such bias can impact on income, opportunity to develop skills and academic profile and overall career progression. Particular verbal and open bias were also reported including sexist jokes, pregnancy-related comments, homophobic comments, liberal use of offensive terms and disparaging comments and stereotypical jokes were commonly experienced.

Equality, diversity and inclusivity (EDI) is important in healthcare. It is established not only in healthcare but also in corporate organisations that diverse teams work better [9]. Highly functioning and effective teams have the potential and ability to deliver improved patient care. Each individual can bring a unique point of view and personal, social and culture experience to the table to enhance patient care. Bias, as defined in the oxford dictionary, is a ‘*strong feeling in favour of or against one group of people often not based on fair judgement’*. Bias may further be categorised as conscious (also described as explicit, describing ones’ awareness of their prejudices) and unconscious (also described as implicit, describing ones’ lack of insight into their own personal attitudes towards certain groups). Diversity bias is a more recently utilised term used to describe individual or systemic bias to those considered in different or more minority groups for example of gender, social or societal groups to those within which we personally identify [7].

It is important to recognise that the practice of surgery is in an exciting period of transition [10]. As many new technologies and techniques are integrating into surgical practice including robotics, artificial intelligence (AI), big data and enhanced imaging, it is also increasingly obvious that the delivery of innovative surgical care is pioneered by an increasingly diverse group of surgeons from non-traditional social backgrounds also with increasing female representation. As a result, it is fair to say that overt bias is beginning to decline. However, the emergence and prevalence of subconscious or implicit bias commonly in the form of micro-aggressions persists [3]. For example, this study identified that while one quarter of respondents felt openly targeted due to their gender, closer to 40% felt they experienced some form of discrimination due to gender bias, so while not all bias is open and obvious it can still occur in a more subtle way.

It is notable that over 60% of the respondents were under 45 years of age. This could be explained by two possible reasons. The first is that this age group is more active in social networks and is more willing to answer this type of surveys. The second could be that young people are more sensitive to equality issues, more aware of potential bias at their workplace, and more willing to report injustices.

Surgery has been traditionally a male dominated specialty and while feminisation of the surgical workforce is increasing, up to 2/3 women still report being deterred from pursuing a career in surgery, experiencing discrimination in the surgical workplace and feeling under-represented in leadership positions [11, 12]. A large systematic review published in early 2021 exploring the unspoken reality of gender bias in surgery identified four broad themes in qualitative analysis, as experiences of women in surgery from 300 female respondents. These included: a general unfavourable work environment with harassment and inability to establish legitimacy; challenges in motherhood, in particular insufficient support from other women, work life balance challenges and negative perceptions of working mothers; a male-dominant culture encouraging female exclusion, requirement to conform to male standards and inequalities in career progression and finally social pressure encompassing generally higher expectation and acceptance of gender stereotypes [11]. While efforts have been made to support the progress of women in surgery, there is still remains a lack of progression to leadership positions and creating a balanced work environment supportive of parenthood [7, 13].

There has been an improvement in the social acceptance of homosexuality and the LGBTQ + community during the last decades. Despite this positive change, many gay, lesbian and bisexual doctors still face many dilemmas, including societal expectations that heterosexuality is the norm [14]. These issues tend to commence in medical school. It has been reported that sexual minority students are more likely to report stress, isolation, verbal insults and harassment or threats, and they are approximately twice as likely to experience depression and related mental health comorbidities [15, 16]. It has previously been reported that surgeons were particularly likely to discourage sexual minorities from entering their specialty [17]. A more recent study provided further evidence that specialties such as surgery are perceived as less inclusive of sexual minorities [18]. This leads to under-representation of LGTBQ + physicians in surgery and a lack of role models. Thus, the global colorectal community needs to consider how this impacts both colleagues and patients. For example, some gay patients may be more open to discuss anal disorders with an LGTBI + colorectal surgeon which could enhance patient care.

In this study a low number of respondents felt they were affected by their sexual orientation at work, which may be impacted by a high number of heterosexual respondents, which was not recorded. However, a higher number of respondents experienced or witnessed attacks, humiliating comments, or a negative work environment because of their or other colleagues’ sexual orientation. This finding contrasts with the results of an independent review on diversity and inclusion for the Royal College of Surgeons of England (RCSEng), which revealed that over 70% of LGBTQ + medics had endured one or more type of harassment or abuse in the previous two years related to their sexual orientation ranging from feeling unable to talk about their private life at one end of the spectrum to homophobic name-calling at the other end [7]. It is important to ensure that the root of this stigma in bias is targeted.

There is a significant lack of data describing surgeons experience of diversity bias due to race or religion from a European perspective. This study identified that 7.5% of Black and minority ethnic (BME) colleagues felt discriminated against due to their race or religion and 12.4% felt they had been openly discriminated against including receiving comments from generalising negative stereotypes and to overt racism. Data from the UK suggest significant bias is experienced by BME colleagues and this can impact on academic and clinical performance and experience. Disparity is observed in professional intercollegiate examinations with BME candidates objectively achieving lower scores [19]. BME colleagues are also less likely to be appointed to specialist surgical training schemes, more likely to experience bullying and harassing behaviour in the workplace and are more likely to be reported to regulatory authorities compared to white/Caucasian colleagues [7]. This is not just in healthcare providers but disparities in provision of healthcare services and clinical outcomes are also observed [20, 21].

While challenges in diversity bias exist both in healthcare and specifically in colorectal surgery, it is important to consider how some of them may be overcome. The increasing current interest by surgical colleges and specialty organisation to focus action on and prioritise EDI is greatly welcomed. The Kennedy Report (2021), published by the RCSEng, called for large scale re-evaluation of how equality and diversity and inclusivity is approach by the surgical community. It highlighted that while a diverse community of surgeons is needed, inclusivity (seeking input and insight from a diverse group) and belonging (making space for everyone and valuing the richness that comes from different perspectives and experiences) are also essential. It is also important to consider the importance of ‘role-modelling’ and ‘visibility initiatives’ so that future leaders in surgery can see themselves and identify with current leaders in their field. Policies with clear procedural application and pathways for raising concerns aligned with actionable outcomes should be an absolute requirement by all medical and surgical colleges and specialty and trainee organisation. It is important that these actions commence as early as medical school (or preferably sooner in life) to ensure that the cultural acceptance of bias is wiped out.

This study has limitations. The response rate is not clearly quantifiable due to the broad dissemination of the questionnaire link through social media platforms. Since no registration was required to respond the survey, double filling could not been prevented. Only three factors (gender, sexual orientation/identity and race or religion) were assessed. These overarching categories of bias were selected as they encompass a significant majority of factors under which bias is experienced, however, the authors recognise that this is not an exhaustive list and that the experience of bias can be multifaceted and relate to an extensive list of personal, professional, and societal factors. Also, some participants who had a more negative experience might have been more willing to respond the survey. While gender variables were recorded, respondents were not asked to describe their sexual orientation or race or religion. Another limitation of this study is that a specific subgroup analysis among different demographic variables of respondents was not performed.

Despite its limitations, this study is the first cross-sectional study aiming to expose biases among surgeons from 37 countries providing a broad, global insight into the diversity bias in colorectal surgery, even though there were limited responses for some countries. In the context of increased reporting about inequalities in career progression, opportunities, and access to surgical training and practice [22, 23], this study provided additional information on EDI in surgery, and identified issues that need urgent action.

## Conclusion

Diversity bias exists in colorectal surgery. The global community of colorectal surgeons must strive to foster a culture of inclusivity and belonging for all colorectal surgeons and colorectal surgeons in training with the aim of learning from and alongside each other. It is also important to strive to support colleagues to overcome workplace clinical dilemmas but also to overcome more personal challenges including bias and discrimination.

## Supplementary Information

Below is the link to the electronic supplementary material.

Supplementary file1 (DOCX 28 KB)

## Declarations

### Human and animals rights

This articles does not contain any studies with human participants or animal performed by any of the authors.
