# Supplementary material for: Diversity bias in colorectal surgery: a global perspective
Source: Updates Surg. 2022 Sep 9;74(6):1915–23. doi: 10.1007/s13304-022-01355-w (PMC9674724; doi:10.1007/s13304-022-01355-w)
Supplement: Supplementary file 1 — Supplementary file1 (DOCX 28 KB) [file 13304_2022_1355_MOESM1_ESM.docx]

**Appendix A: Outline of questionnaire**

1. You are a: *

Man

Woman

I prefer not to answer

1. How old are you? *

25-35 yo

36-45 yo

46-55 yo

56-65 yo

over 65 yo

3.Country where you practice your profession *

4.Professional stage *

Resident

Consultant

5. Have you been personally affected or have you witnessed situations of gender inequality in your current job? *

Yes

No

6. If yes, please answer one of the following options:

My income has been affected

Impact on achieving a promotion or advancing professionally

I have not been invited to give talks, conferences or attend congresses

I have not been able to participate in publishing in scientific journals

Lack of opportunity to develop some type of surgical technique

Other

7. If you selected "other" please provide a brief explanation

8. Have you been personally affected by your sexual orientation in your current job? *

Yes

No

9. If yes, please answer one of the following options:

My income has been affected

Impact on achieving a promotion or advancing professionally

I have not been invited to give talks, conferences or attend congresses

I have not been able to participate in publishing in scientific journals

Lack of opportunity to develop some type of surgical technique

Other

10. If you selected "other" please provide a brief explanation

11. Have you been personally affected by your race or religion in your current job? *

Yes

No

12. If yes, please answer one of the following options:

My income has been affected

Impact on achieving a promotion or advancing professionally

I have not been invited to give talks, conferences or attend congresses

I have not been able to participate in publishing in scientific journals

Lack of opportunity to develop some type of surgical technique

Other

13. If you selected "other" please provide a brief explanation

14. Have you suffered attacks, humiliating comments, or a bad work environment because of your gender? *

Yes

No

15. If yes, please provide a brief explanation

16. Have you suffered or witnessed attacks, humiliating comments, or a bad work environment because of your or other colleagues' sexual orientation? *

Yes

No

17. If yes, please provide a brief explanation

18. Have you suffered or witnessed attacks, humiliating comments, or a bad work environment because of your or other colleagues' race or religion? *

Yes

No

19. If yes, please provide a brief explanation

20. Do you think your work institution or employer guarantees respect in gender equality, sexual orientation or race diversity? *

Yes

No

21. Does your work institution or employer develop programs regarding respect in gender equality, sexual orientation or race diversity? *

Yes

No

22. If yes, briefly explain these programs

23. Are you member of a Scientific Society? (you can choose more than 1 option) *

European Society of Coloproctology (ESCP)

Asociación Española de Coloproctología (AECP)

Società Italiana di Chirurgia Colo-Rettale (SICCR)

Association of Coloproctology of Great Britain and Ireland (ACPGBI)

Association of Surgeons of Great Britain (ASGBI)

Other National Association of Coloproctology or General Surgery

I do not belong to any Scientific Society related to Coloproctology

24. Do you know if the scientific association you belong has any program regarding respect in gender equality, sexual orientation or race diversity?

Yes

No

25. If you wish, please to develop on this.

26. Please type any suggestions or comments.

27. Please write down your email if you want to participate or be contacted for further research in prejudice and diversity bias (optional)

28. By ticking the box you agree for the results of the survey to be presented at the Virtual Annual Conference of the ESCP 2021 and used for potential publication. If you provided an email address, this will not be revealed nor shared with anyone. *

I agree

**Appendix B:** Free-text comments of experience of diversity bias in colorectal surgery and an outline of who can improve each situation of bias described (left sided columns)

| **Gender bias related comments** | **Surgeons** | **Medical School/Hospitals** | **Societies** |
| --- | --- | --- | --- |
| Mandatory on-call duties during pregnancy, against my will | **X** | **X** |  |
| Women are not invited to give talks or conferences as frequent as men | | | **X** |
| *It is easier for male residents to perform surgeries* | **X** | **X** |  |
| Being mistaken for the junior team member due to my gender. *The man must be the surgeon* | **X** |  |  |
| Medical/surgical opinion is not taken into account | **X** | **X** | **X** |
| I felt uncomfortable since some of my colleagues tend to exclude me from their conversations moreover they don’t ask my opinion | **X** |  |  |
| Younger female scrub nurses tend to serve male surgeons better | **X** |  |  |
| The atmosphere clearly changes when a male surgeon enters the OR | **X** |  |  |
| Have to work harder for the same recognition | **X** | **X** |  |
| Gender pre-selection for a specific task | **X** | **X** | **X** |
| Impact on personal relationships with other consultants especially during residency | **X** | **X** |  |
| Lack of trust regarding major abdominal operations | **X** |  |  |
| Women can only be interested in breast or endocrine surgery. Being interested in colorectal surgery is not being appreciated by male colleagues | **X** |  |  |
| Discussions about who is doing what for the day often happen in male changing room between male residents and male consultants - female registrars miss out on these decisions and get less to do… | **X** |  |  |
| Hesitancy to hiring female residents for fear of maternity leave | | **X** |  |
| General lower level of informal opportunity compared to my peers. Lower funding opportunities compared to far less qualified male colleagues |  | **X** | **X** |
| Changes in accorded contracts due to pregnancy |  | **X** |  |
| *Take this surgical marker and go do your eye make up* | **X** | **X** |  |
| I was told that my pregnancy was affecting my job, not taking good decisions in surgery | **X** |  |  |
| Colleagues treat women with paternalism, as if we are residents forever | **X** | **X** | **X** |
| Women are still considered "*not as good as*" a man for major procedures | **X** |  | **X** |
| If there is a choice between a man or a woman the man is still chosen first, just because he is *"one of the boys"* |  | **X** |  |
| Infantilising comments on female patients | **X** |  |  |
| The consultant has a <*ius primae noctis*> over the residents | **X** | **X** | **X** |
| *"you are too ambitious (for a woman)"* | **X** |  |  |
| Male residents can be more relaxed and do a bit less, and they will succeed anyway | **X** | **X** |  |
| Frequently asked if I am a secretary/nurse | **X** |  |  |
| *Are you on your period?* | **X** |  |  |
| *"Mothers should not be surgeons"... "poor kids"* | **X** | **X** | **X** |
| A trainer suggested it’s not worth training me because I’m female and will “*go off to have children*”. | **X** | **X** |  |
| “The head of service does not want to recruit women because of risk of becoming pregnant | | **X** |  |
| Called “the girl” by patients or other colleagues, when my male colleague was referred to as “the doctor” |  |  | **X** |
| Assumed to be more junior and less able than male counterparts and not given as many surgical opportunities comparatively | **X** |  | **X** |
| I was not considered for some kind of surgeries, because surgery needs a “strong man” | **X** |  |  |
| Inappropriate comments in theatre like “*move your boobs out of the way*” | **X** |  |  |
| Male colleagues trying to explain techniques that I know better than them | **X** |  |  |
| Just that some people assume I would do a certain task because it's more suited to a woman according to society norms | **X** |  |  |
| Mobbing and stalking, inappropriate comments about my hair/make up/look, inappropriate physical contacts | **X** | **X** | **X** |
| Some comments about menstrual cycle and lack of sexual activity | **X** |  |  |
| About my appearance, for dating, other colleagues received offensive comments about their weight | **X** |  |  |
| I was told that as a woman I have to choose an outpatient activity or in any case that does not take up the whole day (as it is right that colleagues do) because my priorities must be other or take care of the family. | **X** | **X** |  |
| My consultant bullies me and undermines my clinical judgment with my equal male peers and junior residents | **X** | **X** | **X** |
| I was told that girls are too emotional and was treated differently as my male colleagues when I defended my opinion, even when it was the same one. | **X** |  |  |
| *You don’t need to continue training as you’re a woman and you’ll want a family, you can take a staff grade post* | **X** |  |  |
| I haven’t been given enough opportunity to build up surgical experience even after asking repeatedly | **X** | **X** | **X** |
| *“You’re a blonde*” or *“your abstract won cause you’re the mistress of the chief”* | **X** |  |  |
| Comments that because I'm an Italian woman I won't achieve a permanent job in surgery | **X** |  |  |
| As a woman I have encountered comments such as “*you will probably just get pregnant and leave a gap on the rota*” | **X** |  |  |
| When there was more than one female trainee on the rota “*no one can get pregnant this year or it will really mess with the rota, there aren’t enough men on this rota*” | **X** |  |  |
| Direct quote from a male surgical consultant : ’*You are not bad for a girl!*’ | **X** |  |  |
| *Men work better with men* | **X** |  |  |
| I have been called *saucy* and *feisty* (words not used to describe men) | **X** |  |  |
| Menstrual references, one was pointing out that I must be a typical selfish woman in surgery as I didn’t have kids, my poor husband (I was privately going through fertility challenges and treatment at the time) | **X** | **X** |  |
| Told by a male consultant I was far too confident, as a woman I should be more quiet and less confident | **X** | **X** |  |
|  |  |  |  |
| **Sexuality bias related comments** |  |  |  |
| *“Did you have a change in your metabolism, because of bisexuality?"* | **X** |  |  |
| Homophobic comments are usual in male colleagues, homophobic jokes that no one replies | **X** | **X** | **X** |
| Calling a colleague a "fag" or "slut" because they are homosexual. Implying they are less qualified because of it. | **X** | **X** | **X** |
| Comments undermining capacity of gay surgeons | **X** |  |  |
| Multiple references to not being “safe” with a gay man around | **X** | **X** | **X** |
| depreciatory jokes on homosexual colleagues (calling them weak, not being real men = not worthy to be surgeons) | **X** | **X** | **X** |
| The chief of department openly is speaking homophobic frequently. | **X** | **X** |  |
| After pneumonia hospitalisation: *Have you checked your retroviral status?* | **X** |  |  |
| Bum jokes because I am openly gay | **X** |  |  |
| *Closing with Monocryl is not manly enough, close with clips.* | **X** |  |  |
|  |  |  |  |
| **Race and religion related comments** |  |  |  |
| Employment has been based on my "acceptable" religion | **X** |  | **X** |
| I feel some kind of discrimination for been a Latin-American in Spain. | **X** |  |  |
| I received racist comments from patients as Italian doctor practicing in UE countries. | | | **X** |
| Many senior colleges are speaking xenophobic against coloured students and residents. | **X** | **X** | **X** |
| Offensive comments about my country like “*how come the immigrant treats here?*”, etc |  |  | **X** |
| Immigrants can suffer discrimination and comparisons either with European, National or Local co-workers. | **X** | **X** | **X** |
| Muslim jokes and Sikh jokes | **X** | **X** | **X** |
| I witnessed racist and anti-Muslimism comments. I am actively calling them out, but some continue anyway. | **X** | **X** | **X** |
| Comments like “jokes” regarding my race or country of origin. Sometimes really disrespectful | **X** | **X** | **X** |
| Questioned about ethnicity and that of parents and grandparents and comparisons made to prove that I was of a lower caste from my colleague. | **X** |  |  |
| bad remarks on Muslim colleagues ("*the Al-Qaida guy", "dirty Arab", "they are all the same"*) | **X** | **X** | **X** |
| Belittling of Christian faith publicly in the OR | **X** | **X** | **X** |
| Non-Caucasian doctor colleagues have been subject of racist comments and doubts about their skills from patients | **X** | **X** | **X** |
| I was always expected to perform lower which limit the opportunities I was offered and my work has been shifted to assist rather than key role | **X** |  |  |
| I've witnessed comments on eating habits related to religion/belief. | **X** | **X** |  |
| **n** | **70** | **37** | **28** |
| **%** | **88.61** | **46.84** | **35.44** |
